# Bioelectrical Impedance Analysis–Derived Phase Angle in Initial Medical and Nursing Assessment for Cardiac Rehabilitation: Association with Cardiac Rehabilitation Risk Category and Longitudinal Change During the Program—An Exploratory Prospective Observational Study

**DOI:** 10.3390/jcm15218584

**Published:** 2026-11-04

**Authors:** Francisco José Rodríguez-Velasco, José Alberto Becerra-Mejías, Luis Miguel Lara-Godoy, María Yuste-Domínguez, Julio Carmona-Blesa, Guadalupe Gil-Fernández

**Affiliations:** 1 Department of Nursing, Faculty of Medicine and Health Sciences, University of Extremadura, Av. Elvas s/n, 06006 Badajoz, Spain; jucarmonab@unex.es; 2 Department of Nursing, University Center of Plasencia, University of Extremadura, Av. Virgen del Puerto, n°2, 10600 Plasencia, Spain; 3 Perfusion Nursing Service, Badajoz University Hospital, Extremadura Health Service, Av. Elvas s/n, 06006 Badajoz, Spain; luismiguel.lara@salud-juntaex.es; 4 Cardiology and Cardiac Rehabilitation Service, Badajoz University Hospital, Extremadura Health Service, Av. Elvas s/n, 06006 Badajoz, Spain; maria.yusted@salud-juntaex.es

**Keywords:** phase angle, bioelectrical impedance analysis, cardiac rehabilitation, functional capacity, risk stratification, medicine, nursing, Duke Treadmill Score, CART, sarcopenia, quality of care

## Abstract

**Background**: Cardiac rehabilitation (CR) reduces cardiovascular mortality and improves quality of life in coronary patients. Risk stratification at program onset is essential for individualizing exercise prescription. Phase angle (PhA), derived from bioelectrical impedance analysis, is an index of cell membrane integrity that has emerged as a functional biomarker in various clinical populations. Its utility at the onset of a phase II CR program has not been explored in the Spanish context. This study evaluated the association of PhA with CR risk category, functional capacity and quality of life, its change during the program, and its incremental value over the Duke Treadmill Score (DTS) and clinical variables for risk classification in patients beginning phase II CR. **Methods**: This was an exploratory prospective longitudinal observational study with repeated measures. A total of 109 patients with ischemic heart disease (86.2% male; mean age: 60.2 ± 7.6 years) enrolled in an 8-week phase II CR program (Badajoz, Spain) were included. Phase angle was measured with a multifrequency bioimpedance analyzer (TANITA MC-780MA) under standardized conditions at baseline and program completion. Non-parametric tests (Wilcoxon, Mann–Whitney U, and Spearman correlation), multivariable models adjusted for potential confounders, receiver operating characteristic (ROC) analysis with incremental-value metrics, and a classification and regression tree (CART) evaluated with standard, repeated and nested stratified cross-validation were applied. **Results**: Baseline PhA was significantly higher in the low-risk group than in the high-risk group (5.43 ± 0.65° vs. 4.98 ± 0.81°; U = 1568.5; *p* = 0.002; rank-biserial r = 0.38). PhA correlated positively with the Duke Treadmill Score (r_s = +0.268; *p* = 0.005), physical functioning (r_s = +0.291; *p* = 0.002), vitality (r_s = +0.265; *p* = 0.005) and grip strength (r_s = +0.196; *p* = 0.041), and inversely with age (r_s = −0.288; *p* = 0.002) and the comorbidity index (r_s = −0.198; *p* = 0.039). High-risk status remained associated with lower PhA after adjustment for age, sex, BMI, comorbidity, LVEF, diabetes, revascularization type and time since the event (β = −0.49°; *p* = 0.002). PhA alone showed moderate discrimination (AUC = 0.689; 95% CI: 0.574–0.803); adding PhA to the DTS did not improve discrimination (ΔAUC = +0.004; *p* = 0.748); and a CART model combining DTS, PhA and grip strength (cross-validated accuracy: 85.2%) classified patients identically to the DTS alone. Without the DTS, adding PhA to a clinical model improved model fit and reclassification (likelihood-ratio *p* = 0.003; NRI = 0.64) without a significant AUC gain (+0.067; *p* = 0.106). After 8 weeks, PhA increased slightly (5.27 → 5.33°; *p* = 0.016); the mean change was small (95% CI: 0.01–0.10°) and was not associated with functional changes, and its clinical relevance is uncertain because repeatability data were not available. **Conclusions**: Phase angle is lower in high-risk CR patients independently of age, sex, body composition and comorbidity and correlates weakly to moderately with functional capacity and quality of life. However, it did not add discriminative value to the DTS, and the clinical relevance of its small mean change during the program is uncertain. These exploratory findings show associations only: neither the standalone use of PhA nor its added value over the DTS for risk stratification has been established, and its use for monitoring the response to rehabilitation has not been validated.

## 1. Introduction

Cardiovascular disease (CVD) is the leading cause of mortality in Spain and across European Union countries [1]. Phase II cardiac rehabilitation (CR) is a multicomponent, evidence-based intervention with a Class IA recommendation in clinical guidelines, combining supervised physical training, therapeutic education, and cardiovascular risk factor management over 8–12 weeks [2,3]. The most recent systematic reviews and meta-analyses have confirmed that CR reduces cardiovascular and all-cause mortality and rehospitalizations and significantly improves functional capacity and quality of life in patients with ischemic heart disease [2,3,4,5]. Its cost-effectiveness has also been demonstrated [6]. Despite this evidence, participation in CR in Spain remains among the lowest in Europe, with a historical underutilization of these units [7,8].

Risk stratification at program onset—typically using the Duke Treadmill Score (DTS), the Framingham score, and standardized clinical criteria [8,9]—is essential for individualizing exercise prescription and identifying patients requiring closer monitoring. A deficit of skeletal muscle mass and quality is associated with lower aerobic capacity and a higher risk of mortality in patients with coronary heart disease [10], and the inclusion of resistance training in CR programs has been shown to improve body composition and functional capacity [11,12]. However, current risk stratification does not systematically incorporate cellular quality markers with potentially relevant prognostic impact.

Phase angle (PhA), determined by bioelectrical impedance analysis (BIA), reflects the relationship between the reactance (Xc) and resistance (R) of body tissues and constitutes a direct index of cell membrane integrity and the proportion of active cell mass relative to total body water [13,14]. Higher PhA values indicate greater cellular integrity, better nutritional status, and greater functional muscle mass [13]. Recent systematic reviews have confirmed that PhA at 50 kHz is inversely associated with the presence of cardiovascular disease [15] and with cardiovascular risk factors [16] and constitutes a marker of inflammation in CVD [17]. PhA has also been demonstrated to be a valid indicator of muscle quality [18] and correlates positively with physical activity level in various clinical populations [19].

In patients with ischemic heart disease undergoing CR, recent studies have demonstrated that PhA correlates positively with peak VO_2_ during the late phase of the program [20], whereas in post-myocardial infarction patients completing a 5-week early rehabilitation program, PhA did not change significantly despite favorable changes in other body composition parameters [21]. Skeletal muscle mass, a key determinant of PhA, is inversely associated with mortality and directly associated with aerobic capacity in patients with coronary heart disease [10]. Nevertheless, the utility of PhA as a risk stratification tool at the onset of a phase II CR program has not yet been studied in the Spanish context, nor has its integration with the DTS in a clinical classification model.

Medical and nursing professionals play a central role in baseline functional assessment and follow-up of patients in CR units, and nurse-led or nurse-supported CR has demonstrated improvements in quality of life and biophysiological parameters in coronary patients [22,23,24]. PhA can be obtained easily during the initial medical and nursing evaluation, but whether it provides information relevant to risk stratification or to follow-up during the program has not been established. This exploratory study was designed with the following objectives: (1) to determine whether PhA differs between CR risk groups at baseline assessment, independently of potential confounders; (2) to quantify the correlation between PhA and validated markers of functional capacity and quality of life; (3) to evaluate changes in PhA over the 8-week program as an indicator of longitudinal variation associated with program participation; and (4) to explore, using a clinical decision tree and logistic regression models, whether PhA adds discriminative information to the Duke Treadmill Score and to clinical variables for risk stratification in CR.

## 2. Materials and Methods

### 2.1. Study Design and Registration

A prospective longitudinal observational study with repeated measures was conducted. The participant inclusion period ran from October 2021 to January 2024. The CR program evaluated corresponds to phase II (supervised outpatient), in accordance with the internationally recognized classification of CR phases [25]. The present research is reported following the recommendations of the STROBE statement for observational studies [26]. The CARDYLIFE project (IB20186) was carried out by the GIBIPSO Research Group at the University of Extremadura and was approved by the Bioethics and Biosafety Committee of the University of Extremadura (code 134/2020) and the Research Ethics Committee (REC) of the Badajoz Health Area (reference 19 October 2020), approved in accordance with the Declaration of Helsinki. All participants read and signed written informed consent.

### 2.2. Participants

**Figure 1:**
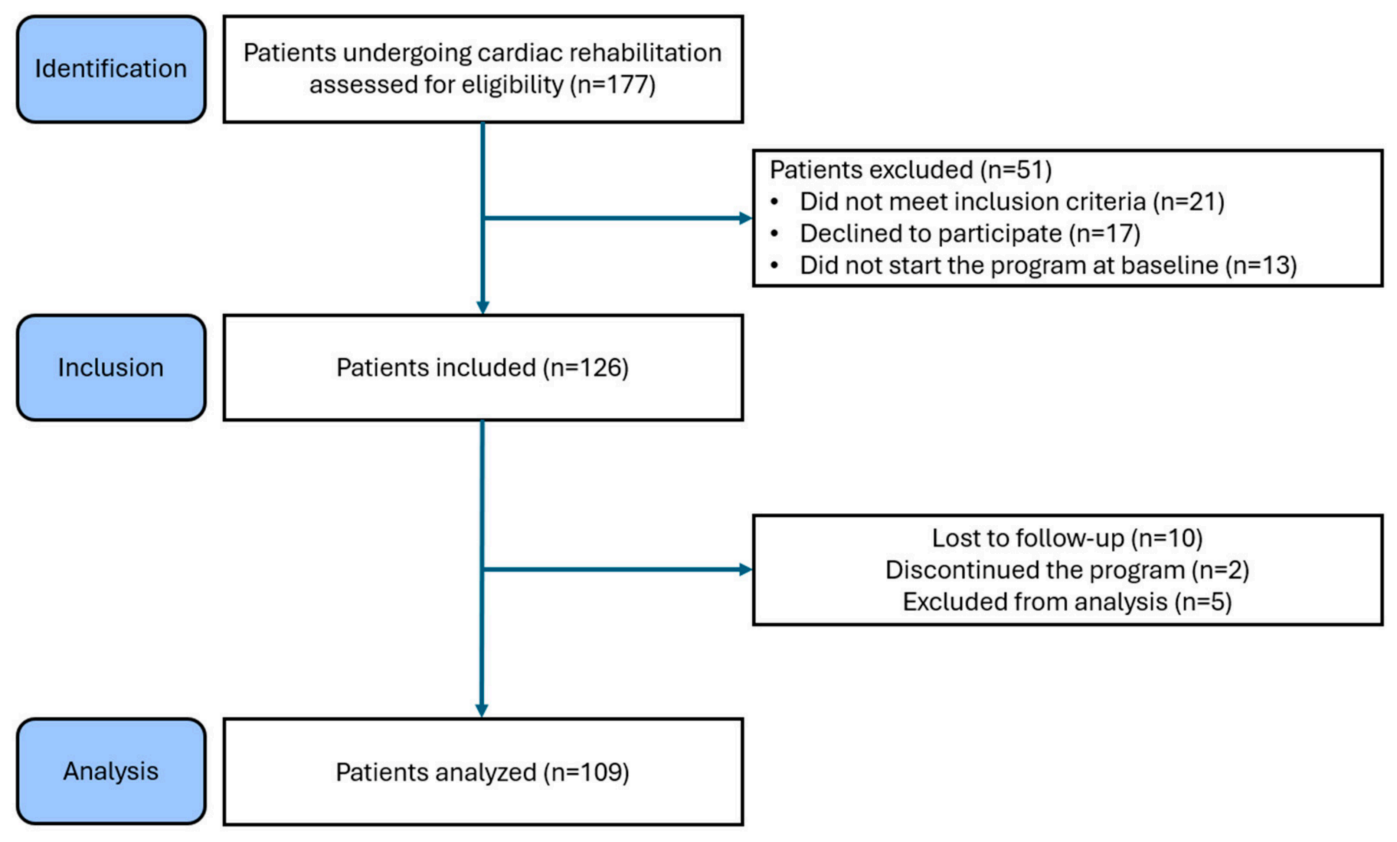
STROBE participant flowchart.

Adult patients (≥18 years) with a confirmed diagnosis of ischemic heart disease referred to the phase II CR program of the Cardiac Prevention and Rehabilitation Unit (CPRU) of the University Hospital of Badajoz (Extremadura Regional Health Service) were included through consecutive, non-probabilistic recruitment, comprising all patients referred during the study period who met the eligibility criteria at the time of baseline assessment. The inclusion criteria were as follows: confirmed diagnosis of acute coronary syndrome (ACS) or chronic ischemic heart disease; capacity to perform supervised physical exercise; and written informed consent. Patients were excluded if they had pacemakers, implantable defibrillation devices or metallic prostheses that could interfere with BIA, active neoplastic disease, or medical contraindications to exercise. Of all patients who enrolled in the program, five were excluded from the definitive analysis due to incomplete data in one or more key study variables, including non-completion of the SF-36 questionnaire at one or both assessment time points. The final analyzable sample comprised 109 participants (Figure 1). In accordance with STROBE recommendations, the characteristics of the 17 excluded participants (across all exclusion stages) were compared with those of the 109 included participants; no systematic differences were identified in age, baseline CR risk category or Charlson Comorbidity Index score, indicating that the pattern of exclusions is unlikely to have introduced material selection bias.

### 2.3. Cardiac Rehabilitation Program

The phase II CR program was outpatient-based and lasted 8 weeks, with supervised physical exercise sessions of 60 min duration, three times per week (24 sessions total). Each session included a warm-up phase (10 min), aerobic training on a cycle ergometer and treadmill with heart rate (HR) monitoring according to the Karvonen HR reserve method (30 min), peripheral muscular resistance training (15 min), and a cool-down phase (5 min). The program was supplemented with group Health Education sessions on cardiovascular risk factor control, dietary therapy, and medication adherence.

### 2.4. Phase Angle Measurement by BIA

Phase angle was determined using the TANITA MC-780MA 8-electrode multifrequency bioelectrical impedance analyzer (TANITA Corporation, Tokyo, Japan), which enables segmental measurement at 5, 50 and 250 kHz. PhA was calculated as PhA = arctan(Xc/R) × (180/π), where Xc is the reactance, and R is the resistance at 50 kHz [14]. The device also provides body mass index (BMI), calculated from the weight measured by the integrated scale and the height entered manually, and estimates visceral fat through proprietary algorithms based on segmental impedance parameters. Measurement conditions were standardized: abstention from intense physical activity in the 24 h prior to measurement, having an empty bladder, and being barefoot [27]. Measurements were performed at baseline assessment (program onset) and at completion of the 8-week program (post-intervention). The same analyzer was used for all measurements, with participants in the standing position as required by the device. The device reports PhA with a resolution of 0.1°. A single measurement was obtained at each time point; duplicate (test–retest) measurements were not performed, so the within-subject reproducibility of PhA could not be estimated directly in this cohort, and changes were interpreted using the indirect approaches described in Section 2.7. The device also provides the extracellular-to-total body water ratio (ECW/TBW), which was used as an index of fluid distribution.

### 2.5. Variables and Assessment Instruments

Functional capacity and risk stratification were assessed using the Duke Treadmill Score (DTS), a validated ergometric index derived from conventional exercise testing according to the formula: DTS = exercise time (min) − (5 × maximum ST-segment deviation [mm]) − (4 × angina index [0 = absent; 1 = non-limiting; 2 = limiting]) [9]. The score ranges from −25 to +15; patients are classified as low risk (DTS ≥ +5), moderate risk (DTS −10 to +4) and high risk (DTS ≤ −11). Health-related quality of life was measured with the SF-36 questionnaire in its validated Spanish version [28], which evaluates 8 dimensions (Physical Functioning, Role Physical, Bodily Pain, General Health, Vitality, Social Functioning, Role Emotional and Mental Health; range: 0–100, where higher scores indicate better health). Global cardiovascular health was assessed with the Fuster-BEWAT index (Blood Pressure, Exercise, Weight, Nutrition/Alimentation, and Tobacco; range: 0–5, where each component is scored 0 or 1 according to fulfilment of predefined cardiovascular health targets, with higher scores indicating better global cardiovascular health) [29]. Comorbidity was quantified using the Charlson Comorbidity Index [30], which assigns weighted scores (1, 2, 3 or 6) to seventeen comorbidity categories; a score of 0 indicates absence of relevant comorbidity, whereas higher scores are associated with greater disease burden and worse long-term prognosis. Peripheral muscle strength was determined by manual grip dynamometry (kg) using a Kern MAP 80K1 dynamometer, recording the mean of three measurements from the dominant hand [31]. Left ventricular function was assessed by left ventricular ejection fraction (LVEF) on echocardiography, classified according to ESC guideline criteria as preserved LVEF (≥50%), mildly reduced LVEF (41–49%) and reduced LVEF (≤40%) [32]. Clinical characteristics recorded at baseline included the index diagnosis, type and completeness of revascularization, number of diseased coronary vessels, presence of arrhythmias, pharmacological treatment and time from the index event to CR onset. Peak oxygen uptake (cardiopulmonary exercise testing) and six-minute walk distance were not available in the study dataset; functional capacity was, therefore, represented by the DTS, grip strength and SF-36 Physical Functioning.

### 2.6. CR Risk Stratification

Cardiovascular risk stratification for the inclusion of patients in the CR program was performed following the guidelines of the European Society of Cardiology [8], classifying the cohort into three mutually exclusive categories: low risk (DTS ≥ 5, preserved LVEF ≥ 50% and strict absence of complex arrhythmias), high risk (DTS < 5, reduced LVEF or complex arrhythmias documented during exercise or at rest) and intermediate or moderate risk, assigned by exclusion to those patients who, while maintaining an absence of complex arrhythmias and a ventricular function not classified as reduced, presented an intermediate combination of the above factors, characterized by a DTS in the intermediate range (−10 to +4) or a mildly but not severely depressed LVEF. This classification is standardly used in the CPRU of the Extremadura Regional Health Service for individualized exercise prescription. PhA was not used to assign the risk category. Because the DTS is one of the criteria used to assign the category, any analysis in which the DTS predicts the category is affected by incorporation bias [33]; the category is not an independent reference standard, and the apparent accuracy of models containing the DTS is inflated. For this reason, the analyses described in Section 2.7 were designed to quantify the information provided by PhA beyond the DTS and beyond clinical variables not involved in the classification. In the analyzed sample, the risk category coincided with the DTS dichotomized at 5 points in 89 of the 101 low- or high-risk patients (88.1%).

### 2.7. Statistical Analysis

Quantitative variables were described as means ± standard deviation (SD) after normality verification with the Shapiro–Wilk test. Given that several variables did not follow a normal distribution, non-parametric tests were applied in all inferential analyses. Comparisons between independent groups (low vs. high CR risk) were performed with the Mann–Whitney U test. Pre–post comparisons (baseline vs. post-intervention) were assessed with the Wilcoxon signed-rank test. Correlations between PhA and functional and quality-of-life variables were calculated with Spearman’s correlation coefficient (r_s). To address multiple simultaneous comparisons, the false discovery rate (FDR) was controlled at q < 0.05 using the Benjamini–Hochberg procedure [34,35]; r^2^ values are reported alongside r_s to contextualize the magnitude of each association. The CART decision tree was fitted with parameters max_depth = 3 and min_samples_leaf = 8, using stratified 5-fold cross-validation for performance estimation. CART hyperparameters (max_depth = 3; min_samples_leaf = 8) were selected a priori to limit model complexity and prevent overfitting given the available sample size, following recommendations for small clinical datasets. A sensitivity analysis using grid search over max_depth ∈ {2,3,4} and min_samples_leaf ∈ {6,8,10} confirmed that these values yielded the most stable cross-validated accuracy. To quantify the independent discriminatory capacity of PhA and the Duke Treadmill Score separately from the CART model, receiver operating characteristic (ROC) curve analysis was performed for the binary comparison between the low-risk and high-risk groups (n = 101; the moderate-risk group [n = 8] was excluded to enable a binary discriminatory comparison, since its small size would have precluded meaningful AUC estimation between distinct risk strata). The area under the ROC curve (AUC) was computed for (1) PhA alone, (2) the Duke Treadmill Score alone, and (3) a combined logistic regression model (Duke + PhA), fitted by L2-regularized logistic regression on standardized predictors (scikit-learn default settings). AUC confidence intervals were estimated by the DeLong method. The optimal discriminatory cut-off for PhA was identified using the Youden index (Youden J = sensitivity + specificity − 1), and the corresponding sensitivity, specificity, positive predictive value (PPV), and negative predictive value (NPV) were calculated. The statistical significance of the difference between AUCs (PhA vs. Duke; combined vs. Duke) was assessed using a bootstrap DeLong test (2000 iterations). The statistical significance level was set at α = 0.05 for all tests. Data processing and analysis were performed using SPSS statistical software (version 25) and JASP software (version 0.97.1). ROC analysis and the machine learning model were carried out using Python 3.10 (SciPy v1.11 and scikit-learn v1.3 libraries). As no a priori sample size calculation was pre-specified for this observational study, statistical power was limited for subgroup comparisons, particularly the moderate-risk group (n = 8) and female participants (n = 15); findings in these subgroups should be considered exploratory and hypothesis-generating. Because the sample size was determined by the number of consecutively recruited, eligible patients during the study period rather than by a pre-specified calculation, a post hoc power analysis was additionally performed for the primary group comparison, the correlation analyses and the paired pre–post comparison, using the effect sizes actually observed and conventional two-independent-samples and Fisher z-transformed correlation formulas based on standard effect-size conventions, implemented in Python (statsmodels v0.15.0); these results are reported in the Discussion (Section 4) to contextualize, rather than substitute for, prospective sample size planning.

Additional analyses were performed during peer review. First, to address potential confounding, baseline PhA was modeled by multiple linear regression with heteroscedasticity-robust (HC3) standard errors, including CR risk group, age, sex, BMI, Charlson Comorbidity Index, LVEF, diabetes under pharmacological treatment, surgical revascularization and time from the index event to CR onset; sensitivity models additionally included ECW/TBW (fluid distribution) or multivessel disease and acute coronary syndrome as the index event (disease severity). Collinearity was checked with variance inflation factors. The association of PhA with high-risk status (low vs. high risk) was also estimated by multivariable logistic regression, and the Spearman correlations were recalculated as partial rank correlations adjusted for age, sex and BMI, with Benjamini–Hochberg correction.

Second, the incremental value of PhA was assessed by comparing nested logistic models: DTS vs. DTS + PhA; a clinical model (age, sex, BMI and the Charlson index) vs. the clinical model + PhA, which does not contain the DTS and is, therefore, not affected by incorporation bias; and the clinical model + DTS vs. the clinical model + DTS + PhA. Comparisons used the likelihood-ratio test (maximum-likelihood models), the bootstrap test for the difference in AUC (2000 resamples), and the category-free net reclassification improvement (NRI) and integrated discrimination improvement (IDI) with bootstrap 95% CIs [36]. Out-of-sample AUC was estimated by repeated stratified 5-fold cross-validation (20 repetitions).

Third, CART performance was reported as accuracy, sensitivity, specificity and balanced accuracy for the high-risk class, together with the pooled out-of-fold confusion matrix, and the original model was compared with CART models using the DTS alone, DTS + PhA, PhA alone, clinical variables (age, sex, BMI, the Charlson index and grip strength) and clinical variables + PhA. In addition to the original stratified 5-fold cross-validation, performance was estimated by repeated stratified 5-fold cross-validation (20 repetitions) and by nested cross-validation, in which hyperparameters were tuned over the same grid within an inner 5-fold loop (10 repetitions), to avoid the optimistic bias of tuning and evaluating on the same folds [37].

Fourth, the change in PhA was additionally described by its distribution, median, Hodges–Lehmann estimator and bootstrap 95% CI of the mean. Test–retest measurements were not available; therefore, measurement error could not be estimated, and no threshold for individual change was derived. The SD of individual changes is reported descriptively; over an 8-week interval it reflects measurement error, biological variation and true heterogeneity of response combined [38,39]. Published within-session reliability data are cited only as contextual information [40]. Associations between the change in PhA (ΔPhA) and changes in DTS, grip strength, SF-36 Physical Functioning and Vitality, and ECW/TBW were assessed with Spearman correlations, and the mean change in PhA was re-estimated after adjustment for changes in ECW/TBW and body weight, given the sensitivity of BIA to fluid status [41]. Finally, probable sarcopenia was operationalized only through the EWGSOP2 grip-strength cut-offs (<27 kg in men and <16 kg in women) [42] because muscle quantity was not assessed with a reference method. These analyses were performed in Python 3.10 (pandas, SciPy v1.15, statsmodels v0.15 and scikit-learn v1.7).

## 3. Results

### 3.1. Baseline Characteristics of the Sample

A total of 109 participants were included (94 males (86.2%); 15 females (13.8%)), with a mean age of 60.2 ± 7.6 years (range: 38–77). The mean body mass index (BMI) was 28.4 ± 4.4 kg/m^2^. The main diagnoses were NSTEMI (36.7%), STEMI (35.8%) and other forms of ischemic heart disease (27.5%). Percutaneous revascularization (PCI) was performed in 58.7% and surgical revascularization (CABG) in 27.5%. The mean time from event to program onset was 131.0 ± 96.4 days. According to CR risk stratification, 61.5% of participants were in the low-risk group (n = 67), 7.3% in the moderate-risk group (n = 8) and 31.2% in the high-risk group (n = 34). Detailed sample characteristics are shown in Table 1. Multivessel disease was present in 51.4% of participants, and 33.0% received pharmacological treatment for diabetes; the median time from the index event to CR onset was 120 days (IQR: 90–150). Compared with the low-risk group, the high-risk group had a higher Charlson index (3.71 ± 1.82 vs. 2.91 ± 1.90; *p* = 0.028) and more frequent surgical revascularization (38.2% vs. 17.9%; *p* = 0.031) and multivessel disease (64.7% vs. 41.8%; *p* = 0.036), whereas age (*p* = 0.080), sex (*p* = 0.254), BMI (*p* = 0.622), LVEF (*p* = 0.207) and time from the event to CR onset (*p* = 0.775) did not differ significantly (Mann–Whitney U or Fisher’s exact test).

### 3.2. Baseline Phase Angle by Risk Group

**Figure 2:**
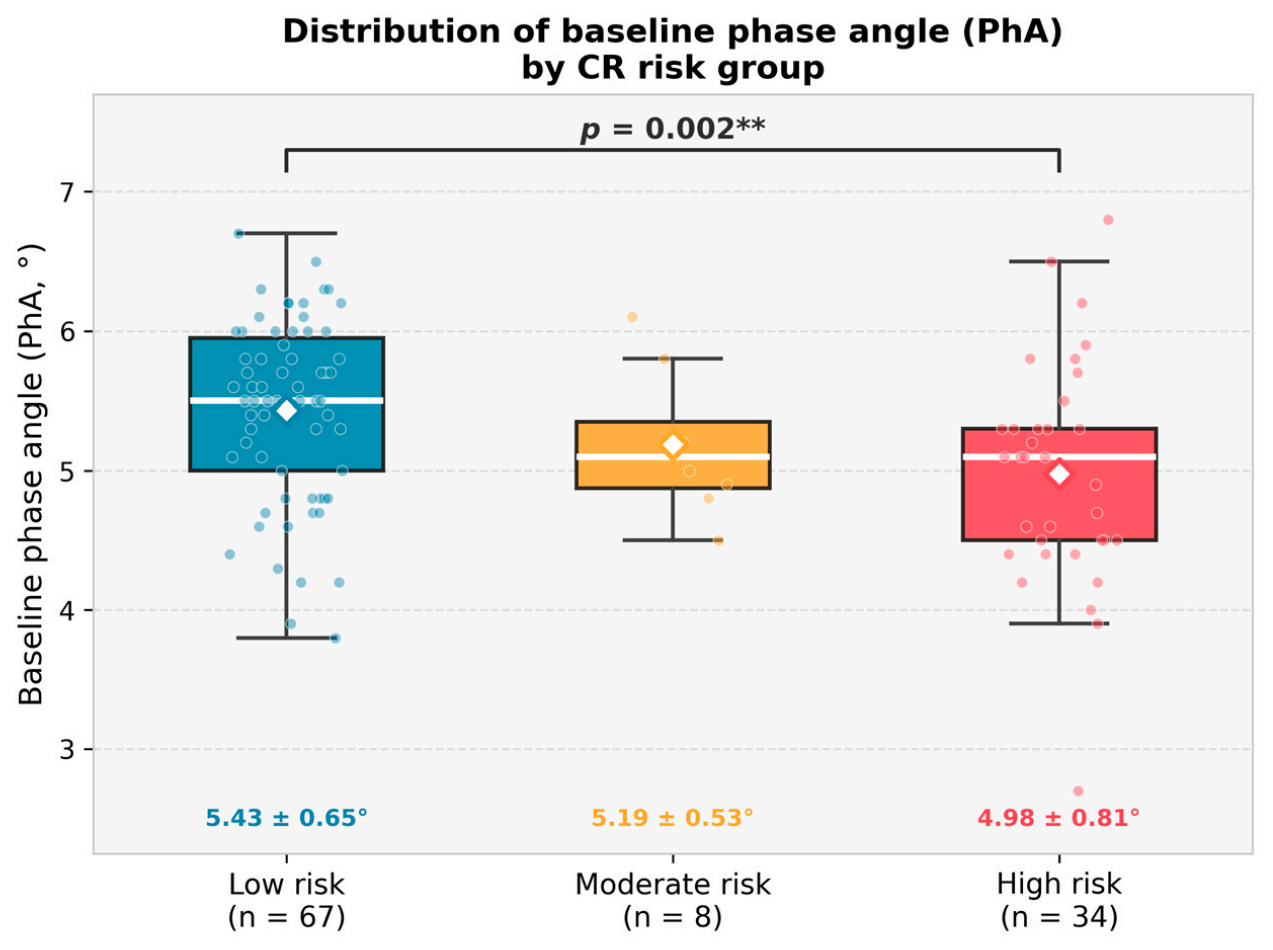
Distribution of baseline phase angle (PhA) by cardiac rehabilitation (CR) risk group. Box plots with individual scatter points are shown. The difference between the low-risk group (5.43 ± 0.65°) and the high-risk group (4.98 ± 0.81°) was statistically significant (U = 1568.5; *p* = 0.002; ** *p* < 0.01; rank-biserial r = 0.38; Mann–Whitney U test). The significance bracket indicates the direct comparison between both extreme groups. Points represent individual participant values.

The baseline PhA of the total sample was 5.27 ± 0.72° (range: 2.7–6.8°). Participants in the low-risk group showed a significantly higher baseline PhA than those in the high-risk group (5.43 ± 0.65° vs. 4.98 ± 0.81°; U = 1568.5; *p* = 0.002; rank-biserial r = 0.38; Figure 2). A significant difference was also observed by sex: males showed a higher baseline PhA than females (5.34 ± 0.69° vs. 4.89 ± 0.80°; *p* = 0.041). At program completion, the difference between risk groups was maintained with greater statistical significance (low risk: 5.52 ± 0.68° vs. high risk: 4.99 ± 0.75°; *p* < 0.001). Group values are detailed in Table 2.

### 3.3. Spearman Correlations Between PhA and Functional Variables

Baseline PhA showed significant correlations with multiple functional capacity and quality-of-life variables (Table 3). All eight associations were retained after Benjamini–Hochberg FDR correction at q < 0.05. The largest observed coefficients were with SF-36 Physical Functioning (r_s = +0.291; r^2^ = 8.5%; *p* = 0.002) and the Duke Treadmill Score (r_s = +0.268; r^2^ = 7.2%; *p* = 0.005). Positive associations were also observed with SF-36 Vitality (r_s = +0.265; r^2^ = 7.0%; *p* = 0.005), SF-36 General Health (r_s = +0.217; r^2^ = 4.7%; *p* = 0.024) and manual grip strength (r_s = +0.196; r^2^ = 3.8%; *p* = 0.041). While statistically significant, all coefficients were weak to moderate in magnitude (r_s range: 0.196–0.291; r^2^ range: 3.8–8.5%), indicating that PhA explains a small proportion of the variance in each outcome variable; clinical relevance should, therefore, be interpreted with caution and not inferred from statistical significance alone [34,35]. Inverse correlations with age (r_s = −0.288; r^2^ = 8.3%; *p* = 0.002) and the Charlson Comorbidity Index (r_s = −0.198; r^2^ = 3.9%; *p* = 0.039) reflect that higher PhA is associated with younger chronological age and lower comorbidity burden. The inverse correlation with the Fuster-BEWAT index (r_s = −0.210; r^2^ = 4.4%; *p* = 0.028) requires a differentiated interpretation: given that the BEWAT assigns higher scores to healthier lifestyle profiles, the negative direction of this correlation indicates that patients with higher baseline PhA paradoxically showed a less favorable lifestyle profile according to this scale; this result, likely influenced by sex and body composition, is discussed in the corresponding section.

### 3.4. Evolution of PhA and Functional Variables Following the Program

After 8 weeks of the CR program, PhA increased significantly in the total sample (5.27 ± 0.72° → 5.33 ± 0.74°; Δ = +0.05° ± 0.23°; *p* = 0.016; d_z = 0.23; Wilcoxon test). This change was of greater magnitude in the low-risk group (low risk: +0.09 ± 0.24°, *p* = 0.014; moderate risk: +0.05° ± 0.03°, *p* = 0.977; high risk: +0.01 ± 0.22°, *p* = 0.299), although the between-group difference in change was not significant (Kruskal–Wallis *p* = 0.246). Significant improvements were simultaneously observed in multiple functional and health variables (Table 4): the Duke score increased by 0.46 points (*p* = 0.005), SF-36 Physical Functioning improved by 4.5 points (*p* = 0.045), SF-36 Vitality improved by 5.6 points (*p* = 0.003) and the Fuster-BEWAT index increased by 0.39 points (*p* < 0.001). Systolic blood pressure (−7.7 mmHg; *p* < 0.001), visceral fat (−0.3 units; *p* < 0.001) and the Framingham risk score (−2.8%; *p* < 0.001) were also significantly reduced. A detailed analysis of the change in PhA is presented in Section 3.9.

### 3.5. Decision Tree Model for Risk Classification

**Figure 3:**
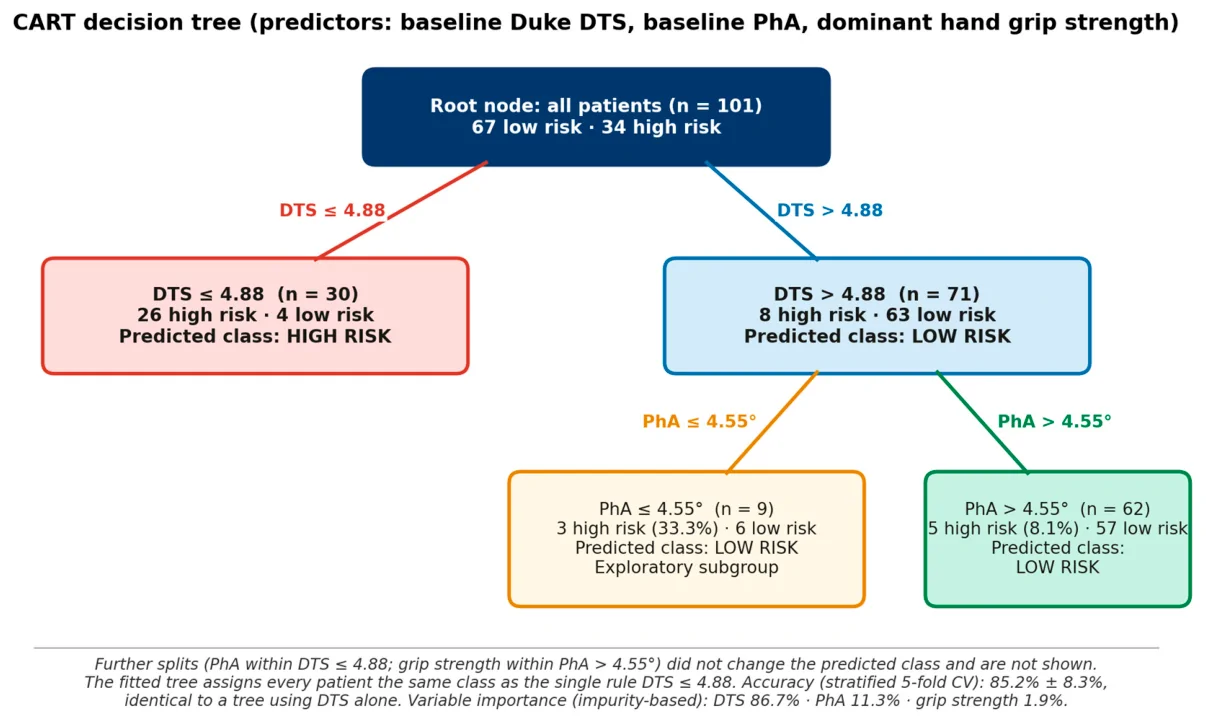
CART decision tree for risk classification in cardiac rehabilitation. The main branch bifurcates according to the Duke Treadmill Score (DTS; cut-off point 4.88 points). Within the DTS > 4.88 branch, baseline phase angle (PhA) ≤ 4.55° separates a small exploratory node (n = 9; 3 high-risk patients) that, like the PhA > 4.55° node, is predicted as low risk; therefore, the fitted tree assigns the same class as the single rule DTS ≤ 4.88. Each node shows the number of patients and its composition by observed risk group (low vs. high risk; n = 101). Overall model accuracy in stratified 5-fold cross-validation was 85.2% (SD ± 8.3%). Variable importance: Duke DTS: 86.7%; PhA: 11.3%; grip strength: 1.9%.

The CART classification tree, including the Duke Treadmill Score, baseline PhA and dominant hand grip strength as predictors, achieved an overall accuracy of 85.2% (SD ± 8.3%) in stratified five-fold cross-validation (Table 5). The dominant variable was the Duke score (relative importance: 86.7%), followed by PhA (11.3%) and grip strength (1.9%). It should be noted that the outcome variable (CR risk group) is largely derived from the Duke score itself, creating a structural overlap between the primary predictor and the classification label; consequently, the reported accuracy reflects predominantly this shared definition rather than purely independent predictive capacity. Individual and incremental discrimination metrics are reported in Section 3.6 and Section 3.8. The main classification rule was Duke ≤ 4.88 → high risk; Duke > 4.88 → low risk. In the branch with Duke > 4.88, PhA ≤ 4.55° separated a small subgroup (n = 9), with a higher proportion of high-risk patients (3/9, 33.3%) than the remaining patients of that branch (5/62; 8.1%); however, because low risk was the majority class in both nodes, this split did not change the predicted class, and the fitted tree classified every patient exactly as the single rule Duke ≤ 4.88. The pooled out-of-fold confusion matrix (with high risk as the positive class) showed 25 true positives, nine false negatives, six false positives and 61 true negatives (sensitivity: 73.5%, specificity: 91.0%, and balanced accuracy: 82.3%). A schematic representation of the tree is shown in Figure 3.

**Table 5:** Characteristics of the CART decision tree model for risk stratification in cardiac rehabilitation.

Parameter	Value
Algorithm	CART (classification and regression tree)
Predictor variables	Baseline Duke DTS; baseline phase angle (PhA-BIA); dominant hand grip strength
Outcome variable	CR risk group (low/high)
Overall accuracy (5-fold CV)	85.2% ± 8.3%
Maximum tree depth	3
Minimum samples per leaf	8
Variable importance	
Baseline Duke DTS	86.7%
Baseline phase angle (PhA)	11.3%
Dominant hand grip strength	1.9%
Main classification rule	Duke ≤ 4.88 → high risk; Duke > 4.88 → low risk
Secondary split (Duke > 4.88 branch)	PhA ≤ 4.55°: n = 9 (3 high/6 low risk); PhA > 4.55°: n = 62 (5 high/57 low risk); predicted class low risk in both nodes (exploratory)
Sensitivity/specificity (high risk)	73.5%/91.0%
Balanced accuracy	82.3%
Pooled out-of-fold confusion matrix	TP 25, FN 9, FP 6, TN 61
CART with Duke DTS alone (5-fold CV)	85.2% ± 8.3% (identical predictions)

Abbreviations: CART, classification and regression tree; CV, cross-validation; DTS, Duke Treadmill Score; PhA, phase angle. Accuracy is expressed as mean ± standard deviation across the 5 cross-validation folds. Sensitivity, specificity, balanced accuracy and the confusion matrix were derived from the pooled out-of-fold predictions, with high risk as the positive class (TP, true positives; FN, false negatives; FP, false positives; TN, true negatives). Repeated and nested cross-validation results are shown in Table 6.

**Table 6:** Performance of CART models for CR risk group classification (low vs. high risk; n = 101; high risk as the positive class).

Predictors	Accuracy, 5-Fold CV (%)	TP/FN/FP/TN	Se/Sp (%)	BAcc (%)	Repeated CV: Acc/BAcc (%)	Nested CV: Acc/BAcc (%)
DTS + PhA + grip (original)	85.2 ± 8.3	25/9/6/61	73.5/91.0	82.3	84.7/80.9	85.1/81.9
DTS alone	85.2 ± 8.3	25/9/6/61	73.5/91.0	82.3	86.0/82.9	85.8/83.0
DTS + PhA	85.2 ± 8.3	25/9/6/61	73.5/91.0	82.3	84.7/80.9	85.1/81.9
PhA alone	69.3 ± 5.0	18/16/15/52	52.9/77.6	65.3	71.4/68.1	70.2/65.9
Clinical	66.2 ± 13.7	16/18/16/51	47.1/76.1	61.6	64.3/56.7	65.0/58.0
Clinical + PhA	77.1 ± 8.3	18/16/8/59	52.9/88.1	70.5	73.8/69.0	70.3/65.1

CART with max_depth = 3 and min_samples_leaf = 8. Accuracy (mean ± SD across folds), confusion matrix, sensitivity (Se), specificity (Sp) and balanced accuracy (BAcc) from the original stratified 5-fold cross-validation (pooled out-of-fold predictions). Repeated CV: mean of 20 repetitions of shuffled stratified 5-fold cross-validation with fixed hyperparameters. Nested CV: hyperparameters (max_depth ∈ {2,3,4}; min_samples_leaf ∈ {6,8,10}) tuned by inner 5-fold cross-validation maximizing balanced accuracy; mean of 10 repetitions. Clinical: age, sex, BMI, Charlson Comorbidity Index and dominant hand grip strength. Majority-class accuracy: 66.3%. Abbreviations: Acc, accuracy; CART, classification and regression tree; CV, cross-validation; DTS, Duke Treadmill Score; FN, false negative; FP, false positive; PhA, phase angle; TN, true negative; TP, true positive.

### 3.6. Discriminatory Capacity of PhA for CR Risk Classification: ROC Analysis

**Figure 4:**
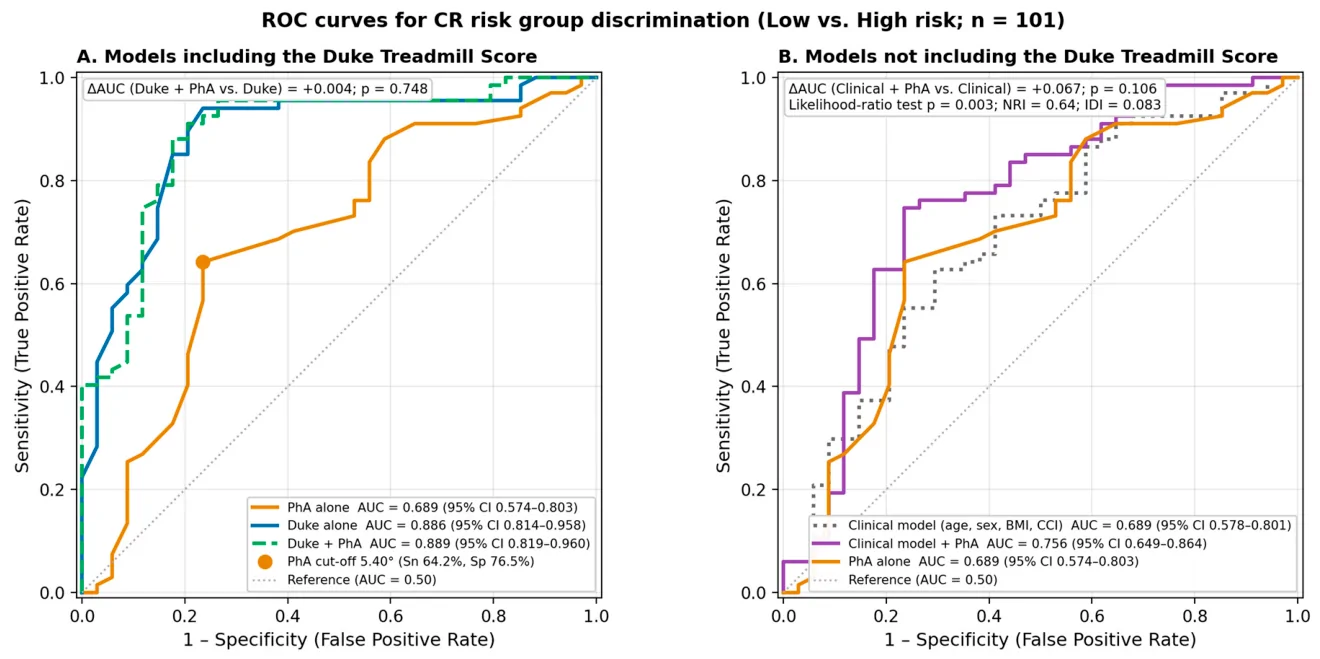
ROC curves for discrimination of CR risk groups (low vs. high risk; n = 101). (**A**) Models including the Duke Treadmill Score: phase angle (PhA) alone (orange; AUC = 0.689; 95% CI: 0.574–0.803), with the optimal Youden cut-off (orange dot; 5.40°; sensitivity: 64.2%, specificity: 76.5%, low risk as the positive class); Duke Treadmill Score alone (blue; AUC = 0.886; 95% CI: 0.814–0.958); and combined Duke + PhA logistic model (green dashed; AUC = 0.889; 95% CI: 0.819–0.960). The increment in AUC from adding PhA to Duke was 0.004 (bootstrap test: *p* = 0.748). (**B**) Models not including the Duke Treadmill Score: clinical model with age, sex, BMI and Charlson Comorbidity Index (CCI) (grey dotted; AUC = 0.689; 95% CI: 0.578–0.801) and clinical model + PhA (purple; AUC = 0.756; 95% CI: 0.649–0.864; ΔAUC = +0.067, bootstrap *p* = 0.106; likelihood-ratio test *p* = 0.003); PhA alone is shown for reference. Grey dotted diagonal: reference (AUC = 0.50). AUC 95% CIs by the DeLong method.

To directly address the independent discriminatory value of PhA—separately from the CART model—an ROC analysis was performed on the binary comparison between the low-risk group (n = 67) and the high-risk group (n = 34), excluding the moderate-risk group (n = 8; n = 101 for this analysis). PhA alone achieved an AUC of 0.689 (95% CI: 0.574–0.803), indicating moderate but statistically significant discrimination above chance. At the optimal Youden cut-off of 5.40°, sensitivity was 64.2%, and specificity was 76.5% (PPV = 84.3%; NPV = 52.0%). Duke alone achieved an AUC of 0.886 (95% CI: 0.814–0.958); the difference in AUC between PhA and Duke was significant (ΔAUC = −0.197; bootstrap DeLong test: z = −3.06; *p* = 0.002). The combined logistic model (Duke + PhA) yielded an AUC of 0.889 (95% CI: 0.819–0.960), an increment of 0.004 over Duke alone that did not reach statistical significance (*p* = 0.748). These results indicate that PhA has moderate standalone discriminatory ability and does not significantly improve overall discrimination when added to the Duke score in a logistic model. At the post hoc Youden cut-off, the DTS alone showed a sensitivity for the high-risk group of 76.5% and a specificity of 94.0% (balanced accuracy: 85.3%), and the Duke + PhA model showed 79.4% and 91.0% (balanced accuracy: 85.2%). The cross-validated AUCs were 0.675 for PhA alone, 0.878 for Duke alone and 0.874 for Duke + PhA (Table 7). Summary metrics are shown in Table 8 and the ROC curves in Figure 4.

### 3.7. Adjustment for Potential Confounders

In multivariable linear regression (n = 105 with available LVEF), high-risk group membership remained independently associated with lower baseline PhA (β = −0.49°; 95% CI: −0.79 to −0.18; *p* = 0.002), together with older age, female sex and lower BMI, whereas the Charlson index, LVEF, diabetes, surgical revascularization and time from the event were not significantly associated (R^2^ = 0.30; variance inflation factors ≤ 2.03; Table 9). The association persisted after additional adjustment for ECW/TBW (β = −0.32°; *p* = 0.015) or for multivessel disease and acute coronary syndrome as the index event (β = −0.50°; *p* = 0.001). In logistic regression (low vs. high risk; n = 101), each 1° higher PhA was associated with lower odds of high-risk classification after adjustment for age, sex, BMI and Charlson index (OR: 0.34; 95% CI: 0.16–0.74; *p* = 0.006), and with the extended adjustment set of Table 9 (OR: 0.27; 95% CI: 0.11–0.64; *p* = 0.003; n = 98), although the events-per-variable ratio of the latter model was low (3.8). The difference in PhA between low- and high-risk patients was observed in both sexes (men: 5.45° vs. 5.13°, *p* = 0.033; women: 5.32° vs. 4.39°, *p* = 0.010).

After adjustment for age, sex and BMI, the partial correlations of PhA with SF-36 Physical Functioning (r = 0.342; FDR-adjusted *p* = 0.002) and Vitality (r = 0.309; FDR-adjusted *p* = 0.004) remained significant, those with SF-36 General Health (r = 0.207) and the DTS (r = 0.205) were attenuated (unadjusted *p* = 0.033 and 0.035; FDR-adjusted *p* = 0.061 for both), and those with grip strength (r = 0.057), the Charlson index (r = −0.038) and the Fuster-BEWAT score (r = −0.091) were no longer significant. In a multivariable model of the baseline DTS including the covariates of Table 9, PhA was not independently associated with the DTS (β = 0.82 points per degree; 95% CI: −0.20 to 1.83; *p* = 0.114). Probable sarcopenia according to EWGSOP2 grip-strength cut-offs was present in 13 participants (11.9%), whose PhA did not differ significantly from that of the remaining participants (5.08 ± 0.46° vs. 5.30 ± 0.74°; *p* = 0.198).

### 3.8. Incremental Value of PhA and Cross-Validated Performance of the Classification Models

Adding PhA to the DTS improved model fit (likelihood-ratio test *p* = 0.028; OR per 1° = 0.42; 95% CI: 0.18–0.94) but not discrimination (ΔAUC = +0.004; *p* = 0.748; NRI = 0.34, 95% CI: −0.07 to 1.03; IDI = 0.029, 95% CI: −0.0004 to 0.135) (Table 7). In the clinical model without the DTS, PhA was independently associated with high-risk status (OR per 1° = 0.34; 95% CI: 0.16–0.74; *p* = 0.006) and improved model fit and reclassification (likelihood-ratio test *p* = 0.003; NRI = 0.64, 95% CI: 0.12 to 1.07; IDI = 0.083, 95% CI: 0.008 to 0.209), whereas the increase in AUC (0.689 to 0.756; ΔAUC = +0.067; *p* = 0.106) was not statistically significant, and cross-validated discrimination remained modest (CV-AUC: 0.622 to 0.685). Similar results were obtained when PhA was added to the clinical model + DTS (likelihood-ratio test *p* = 0.003; ΔAUC = +0.026; *p* = 0.117), although this model had a low events-per-variable ratio (34 events; six predictors).

CART performance is shown in Table 6. The original model (DTS, PhA and grip strength) and the CART using the DTS alone produced identical out-of-fold predictions (accuracy: 85.2% ± 8.3%; balanced accuracy: 82.3%). In repeated cross-validation, the DTS-alone tree performed at least as well as the trees including PhA (balanced accuracy: 82.9% vs. 80.9%), and nested cross-validation yielded similar estimates (83.0% vs. 81.9%). Without the DTS, adding PhA to clinical variables increased CART balanced accuracy from 56.7% to 69.0% in repeated cross-validation (nested: 58.0% to 65.1%), but performance remained well below that of the DTS-based models. For reference, the simple rule DTS < 5 reproduced the risk category with an apparent accuracy of 88.1% (sensitivity: 76.5%; specificity: 94.0%), and the majority-class rate was 66.3%.

### 3.9. Characteristics of the Longitudinal Change in PhA

PhA increased in 57 participants (52.3%), was unchanged in 20 (18.3%) and decreased in 32 (29.4%); because the device reports PhA in steps of 0.1°, individual changes were in multiples of 0.1° (range: −0.8° to +0.6°). The median change was +0.1° (IQR: −0.1° to +0.2°), the Hodges–Lehmann estimate was +0.05°, and the bootstrap 95% CI of the mean change was 0.01° to 0.10° (paired t-test *p* = 0.018). The SD of individual changes was 0.23°; because this variability combines measurement error, biological variation and true differences in response, and no test–retest data were available, it was not used to classify individual changes. ΔPhA was inversely correlated with the change in ECW/TBW (r_s = −0.412; *p* < 0.001); after adjustment for changes in ECW/TBW and body weight, the mean change in PhA was +0.046° (95% CI: 0.004 to 0.088; *p* = 0.030). ΔPhA was not significantly correlated with changes in the DTS (r_s = −0.159; *p* = 0.099), grip strength (r_s = +0.183; *p* = 0.056), SF-36 Physical Functioning (r_s = +0.154; *p* = 0.110) or SF-36 Vitality (r_s = +0.181; *p* = 0.060). Grip strength did not change significantly over the program (+0.3 ± 4.1 kg; *p* = 0.520).

## 4. Discussion

This exploratory study shows that PhA obtained by BIA is significantly lower in high-risk than in low-risk patients at the onset of a phase II CR program (low risk: 5.43° vs. high risk: 4.98°; *p* = 0.002), and that this difference persists after adjustment for age, sex, BMI, comorbidity, LVEF, diabetes, revascularization type and time since the event, as well as in sex-stratified analyses. This finding is consistent with prior evidence associating reduced PhA values with the presence of cardiovascular diseases [15,16] and with inflammatory markers in CVD [17]. The most recent meta-analyses confirm that PhA at 50 kHz is significantly lower in CVD patients compared with healthy controls [15] and that it is inversely associated with cardiovascular risk factors such as hypertension, dyslipidemia, and obesity [16]. Higher-risk CR patients present greater comorbidity and lower functional capacity, which converge in lower cellular integrity, as reflected by PhA [14,21].

The Spearman correlations between baseline PhA and functional capacity indicators—DTS (r_s = 0.268), SF-36 Physical Functioning (r_s = 0.291) and Vitality (r_s = 0.265)—are consistent with the recent literature. Onishi et al. [20] demonstrated in patients in the late phase of CR that PhA correlates with peak VO_2_, supporting that it captures dimensions of aerobic capacity not directly assessed by routine clinical indicators, consistent with the distinction between functional capacity (maximal potential) and functional performance (actual behavior) proposed by Leidy [43]. In that study of 107 patients, the correlation between PhA and peak VO_2_ measured by cardiopulmonary exercise testing was strong (r = 0.603 after CR), and PhA remained an independent predictor of peak VO_2_ after adjustment for age, sex, grip strength, skeletal muscle index, nutritional status and NT-proBNP [20]. The weaker associations observed in our cohort (r_s: 0.20–0.29) probably reflect, at least in part, that the DTS is a composite prognostic score that includes ST-segment deviation and angina in addition to exercise time, rather than a direct measure of aerobic capacity; peak VO_2_ and six-minute walk distance were not available in our study. After adjustment for age, sex and BMI, the associations of PhA with SF-36 Physical Functioning and Vitality persisted, whereas those with the DTS and General Health were attenuated, and that with grip strength disappeared, indicating that part of the crude correlations reflects shared determinants of PhA and function. The positive association with grip strength is consistent with evidence identifying PhA as a biomarker of muscle quality [18] and with its correlation with physical activity level [19]. Notably, the EWGSOP2 revised European consensus on sarcopenia operationally defines this condition by combining muscle strength—assessed by grip dynamometry—with muscle mass or quality indices [42]. However, sarcopenia was not diagnosed in this study with a validated EWGSOP2 assessment including muscle quantity measured by a reference method; the association between PhA and grip strength did not persist after adjustment for age, sex and BMI; and participants with probable sarcopenia according to EWGSOP2 grip-strength cut-offs did not have significantly lower PhA. Therefore, any link between PhA and sarcopenia in this population—including the interpretation of the exploratory PhA ≤ 4.55° node of the CART model—should be regarded as a hypothesis to be tested rather than a finding of this study. The inverse correlation with age and the Charlson Comorbidity Index reflects the loss of cellular integrity associated with aging and comorbidity, a phenomenon also described in patients with COPD [44] and on hemodialysis [45]. Nichols et al. [10] demonstrated that skeletal muscle mass—the main determinant of PhA—is inversely associated with mortality and directly associated with aerobic capacity in coronary patients.

The inverse correlation between baseline PhA and the Fuster-BEWAT index (r_s = −0.210; *p* = 0.028) warrants specific reflection. Given that the BEWAT assigns higher scores to healthier lifestyle profiles, the negative direction of this correlation implies that, in the study cohort, patients with higher baseline PhA tended to show poorer lifestyle habits according to this tool. This apparent paradox can be explained by the influence of sex on PhA: in the literature, males systematically present higher PhA values than females, related to their greater skeletal muscle mass [46,47]; in a cohort with a large male predominance (86.2%) such as the one studied, males—who globally show higher PhA—may concentrate lower BEWAT scores on items such as weight or tobacco consumption. Consistently, Ferraz et al. [48] demonstrated in outpatient cardiac patients that PhA is inversely associated with visceral fat and estimated cardiovascular risk, evidencing that interpretation of the PhA–lifestyle relationship requires adjustment for body composition and sex. Future studies should examine this association stratified by sex and body composition to determine whether the inverse PhA-BEWAT correlation persists independently of differences in body composition between sexes. In the present data, the inverse correlation persisted among men (r_s = −0.232; *p* = 0.025; women: r_s = −0.167; *p* = 0.551, n = 15) but was no longer significant after adjustment for age, sex and BMI (partial r = −0.091; *p* = 0.352), suggesting that age and body size, rather than sex alone, account for most of this association.

The statistically significant increase in PhA after 8 weeks of CR (Δ = +0.05° ± 0.23°; *p* = 0.016; d_z = 0.23) should be compared critically with previous longitudinal studies. Onishi et al. [20] reported larger increases during the late-recovery phase CR (from 4.69° to 4.89° in men and from 4.06° to 4.47° in women) in patients with lower baseline values than ours (5.27°). Conversely, Ślązak et al. [21], using the same analyzer model (TANITA MC-780) in 55 post-myocardial infarction patients who completed a 5-week early rehabilitation program, found no significant change in PhA despite reductions in visceral fat and ECW/TBW and attributed this to the good baseline condition of their patients and the short observation period. Shibata et al. [49] described a progressive recovery of PhA over six months after cardiovascular surgery, in which grip strength and nutritional indices were key correlates, and Onishi et al. [50] recently reported that PhA at CR completion—but neither baseline PhA nor its change—independently predicted unplanned readmission. Taken together, these data suggest that the magnitude of change in PhA depends on baseline status, program duration and population, and that the value attained may be more informative than the change itself. In our cohort, the mean change must be interpreted with caution. Because a group mean is estimated from many measurements, it can legitimately be smaller than the display resolution of the device (0.1°), and the resolution alone does not invalidate the observed change. The main limitations lie elsewhere. First, no test–retest measurements were obtained, so the contribution of measurement error to individual changes cannot be quantified; published within-session minimal detectable changes for standing octapolar analyzers at 50 kHz (0.11–0.14°) [40] are not directly transferable to an 8-week interval, which also includes biological variation. Second, the clinical relevance of the change is uncertain (see below). In addition, ΔPhA was strongly related to the change in ECW/TBW, in keeping with the sensitivity of BIA to fluid distribution [41], although a small adjusted increase persisted; ΔPhA was not significantly associated with changes in the DTS, grip strength or SF-36 scores; and, in the absence of a control group, the change cannot be attributed to the CR intervention. The concomitant improvements in SF-36 Physical Functioning (+4.5 points), Vitality (+5.6 points), DTS (+0.46 points) and BEWAT (+0.39 points), together with the favorable lipid dynamics described in similar programs [51], are consistent with temporal patterns of change reported in controlled CR efficacy studies [2,3,52]; whether the small parallel change in PhA reflects a physiological adaptation requires confirmation in controlled designs with repeated baseline measurements. Although statistically significant, the magnitude of the observed change (Δ = +0.05°; SD = 0.23°) falls below the conventional smallest worthwhile change threshold (0.2 × SD_baseline = 0.14°). No minimal clinically important difference (MCID) has been established for PhA in patients beginning cardiac rehabilitation; accordingly, the clinical relevance of this change cannot be determined with the current evidence and should be interpreted with caution.

The CART decision tree combining the Duke Treadmill Score, PhA and grip strength achieved an accuracy of 85.2% in five-fold cross-validation. As noted, an inherent limitation of this figure is the structural circularity between the primary predictor (Duke) and the outcome label (CR risk group). To address this directly, ROC analyses were performed for each predictor individually and for a combined logistic model. PhA alone achieved an AUC of 0.689 (95% CI: 0.574–0.803)—moderate but statistically significant discrimination above chance—compared with an AUC of 0.886 (95% CI: 0.814–0.958) for Duke alone (ΔAUC = −0.197; *p* = 0.002). The combined Duke + PhA logistic model achieved an AUC of 0.889 (95% CI: 0.819–0.960), an increment of only 0.004 over Duke alone that was not statistically significant (*p* = 0.748). These findings indicate that PhA has moderate standalone discriminatory ability and does not significantly improve overall AUC when added to the Duke score in logistic regression. Moreover, the CART split on PhA within the Duke > 4.88 branch did not change any predicted class: the fitted tree classified all patients exactly as the single rule Duke ≤ 4.88, and its cross-validated performance was identical to, or slightly lower than, that of a tree using the DTS alone (Table 6). The PhA ≤ 4.55° node included only nine patients (three of them high-risk) and must be regarded as exploratory. The optimal Youden cut-off for PhA in the binary ROC analysis (5.40°) differs from the CART-derived cut-off (4.55°), reflecting that the latter applies only within the Duke > 4.88 branch. To address the incorporation bias inherent to the DTS, PhA was also evaluated in models that did not include the DTS: adding PhA to age, sex, BMI and Charlson index improved model fit and reclassification (likelihood-ratio *p* = 0.003; NRI: 0.64, 95% CI: 0.12–1.07; IDI: 0.083, 95% CI: 0.008–0.209) [36], but the AUC gain (+0.067) was not significant and the cross-validated AUC remained modest (0.685). Likewise, adding PhA to the DTS improved model fit (likelihood-ratio *p* = 0.028) without improving discrimination. Overall, PhA carries information on the risk category that is not captured by age, sex, BMI and comorbidity, but this information does not translate into a clinically useful gain in classification over the DTS, and its added value for risk stratification is not established by the present data. The PhA cut-off and grip strength contribution require prospective validation in external cohorts.

From a clinical practice perspective—both medical and nursing—PhA measurement has some practical features that would facilitate its further evaluation in CR units: (1) BIA measurement is fast and non-invasive and can be performed by trained medical or nursing staff, although device- and protocol-specific reproducibility should be established locally; (2) segmental multifrequency analysis simultaneously provides body composition, impedance and PhA data at marginal cost relative to routine measurements; and (3) PhA values can be recorded in the unit’s clinical information system to build the longitudinal datasets needed for future validation. Within Donabedian’s quality-of-care model (structure–process–outcome) [53], incorporating PhA-BIA measurement into the initial medical and nursing assessment could constitute a process indicator: the structural element is the BIA device already present in the CR unit; the process element is the standardized medical and nursing assessment integrating PhA alongside functional capacity measures; however, no outcome benefit, such as improved risk stratification, has been demonstrated by the present data. Nurse-led or nurse-supported CR has demonstrated improvements in quality of life and biophysiological outcomes in coronary patients [22,23,24]; whether incorporating PhA into their assessment protocols adds clinical value remains to be established. Previous studies in Spain have shown that technological interventions in CR improve adherence and clinical outcomes [7], which supports the feasibility of evaluating new assessment tools such as PhA in this setting. Contemporary CR programs conceive prognostic stratification as a continuous process, with periodic assessment of outcomes by multidisciplinary teams that include nurses [54]; within this framework, PhA could be considered a complementary descriptor of the patient’s status rather than a stand-alone criterion for risk classification.

Several limitations of this study should be acknowledged. First, as a longitudinal observational study with repeated measures without a control group, causal relationships cannot be established; the absence of a concurrent control group precludes exclusively attributing the observed changes in PhA to the program. Second, no formal a priori sample size calculation was performed for this observational analysis; the sample size was instead determined by the number of consecutively recruited, eligible patients during the study period. To contextualize this limitation, a post hoc power analysis was conducted using the effect sizes actually observed [55]. For the primary comparison (baseline PhA, low-risk vs. high-risk groups), the observed effect size (Cohen’s d = 0.64, consistent with the reported rank-biserial r = 0.38) corresponds to a post hoc power of 85% with the sample available for this comparison (n = 67 vs. n = 34; α = 0.05, two-sided); the sample size that would have been required a priori to detect this effect with 80% power was approximately 80 participants (40 per group under 1:1 allocation), fewer than the 101 patients analyzed. The two strongest correlations were likewise adequately powered (SF-36 Physical Functioning, r_s = 0.291, power = 87%; Duke Treadmill Score, r_s = 0.268, power = 81%). In contrast, this study was underpowered for the weakest significant correlation (dominant hand grip strength, r_s = 0.196, power = 53%; approximately 202 participants would have been required for 80% power) and for the paired pre–post change in PhA (d_z = 0.23, power = 66%; approximately 150 participants would have been required for 80% power); these two findings should, therefore, be interpreted with particular caution and require replication in adequately powered samples. Statistical power remained limited for subgroup comparisons, particularly the moderate-risk group (n = 8) and female participants (n = 15); analyses in these subgroups should be regarded as exploratory and hypothesis-generating. Third, the eight Spearman correlations were performed simultaneously; although all associations were retained after Benjamini–Hochberg FDR correction at q < 0.05, several *p*-values were close to the conventional threshold, and the coefficients were of weak-to-moderate magnitude (r_s: 0.196–0.291; r^2^: 3.8–8.5%), limiting their direct clinical applicability [34,35]. Moreover, the correlations with grip strength, the Charlson index and the Fuster-BEWAT score were no longer significant after adjustment for age, sex and BMI. Fourth, the male predominance (86.2%) restricts the generalizability of findings to women with ischemic heart disease. Fifth, the CR risk category is partly defined by the DTS and is not an independent reference standard; models including the DTS are, therefore, affected by incorporation bias, which inflates their apparent accuracy, and models without the DTS showed only modest cross-validated discrimination. The model was internally validated only, and independent external validation is required before clinical adoption. Sixth, the PhA cut-off of 4.55° identified by the model is based on only nine patients and requires prospective validation. Finally, no test–retest measurements of PhA were performed, so device- and protocol-specific reproducibility could not be quantified directly; peak VO_2_ and six-minute walk distance were not available; sarcopenia was not assessed with complete EWGSOP2 criteria; residual confounding by unmeasured factors (e.g., inflammation, nutritional intake or fluid status at the time of measurement) cannot be excluded; and some multivariable logistic models had a low number of events per variable, so their estimates may be unstable.

## 5. Conclusions

Phase angle measured by bioelectrical impedance analysis was lower in high-risk patients at the onset of a phase II cardiac rehabilitation program, independently of age, sex, body composition, comorbidity and other clinical variables, and correlated weakly to moderately with indicators of functional capacity and quality of life. However, PhA did not improve discrimination when added to the Duke Treadmill Score: the CART model including PhA classified patients identically to the Duke score alone, whose contribution is inflated because it also defines the risk category. In models without the Duke score, PhA improved model fit and reclassification, but cross-validated discrimination remained modest. The small mean increase in PhA observed over 8 weeks was not associated with functional changes; in the absence of repeatability data, its clinical relevance is uncertain, and, without a control group, it cannot be attributed to the program. Therefore, this exploratory study supports associations between PhA and patient characteristics relevant to cardiac rehabilitation; neither the standalone use of PhA nor its added value over the Duke Treadmill Score for risk stratification has been established, and its use for monitoring the response to rehabilitation has not been validated. PhA may be recorded as a complementary descriptor in the initial medical and nursing assessment while its clinical utility is evaluated in controlled studies with reproducibility assessment and external validation.

## Figures and Tables

**Table 1:** Baseline characteristics of the sample (N = 109).

Variable	n (%)	Mean ± SD	Range
Sample size	109 (100%)	—	—
Male sex	94 (86.2%)	—	—
Age (years)	—	60.2 ± 7.6	38–77
BMI (kg/m^2^)	—	28.4 ± 4.4	21.8–48.4
Baseline LVEF (%)	—	51.0 ± 4.8	40–67
Charlson Comorbidity Index	—	3.15 ± 1.86	0–9
Number of medications	—	7.3 ± 2.3	2–14
Time from event to CR onset (days)	—	131.0 ± 96.4	4–720
Main diagnosis			
NSTEMI	40 (36.7%)	—	—
STEMI	39 (35.8%)	—	—
Other forms of ischemic heart disease	30 (27.5%)	—	—
Type of revascularization			
Percutaneous (PCI)	64 (58.7%)	—	—
Surgical (CABG)	30 (27.5%)	—	—
No revascularization	15 (13.8%)	—	—
CR risk group			
Low risk	67 (61.5%)	—	—
Moderate risk	8 (7.3%)	—	—
High risk	34 (31.2%)	—	—
Additional clinical characteristics			
Time from event to CR onset, median (IQR), days	—	120 (90–150)	—
LVEF 41–49% (mildly reduced) ^1^	9 (8.3%)	—	—
LVEF ≤ 40% (reduced) ^1^	2 (1.8%)	—	—
Multivessel disease (≥2 vessels)	56 (51.4%)	—	—
Incomplete revascularization	10 (9.2%)	—	—
Simple arrhythmias at baseline ^2^	35 (32.1%)	—	—
Diabetes (pharmacologically treated)	36 (33.0%)	—	—
Insulin therapy	6 (5.5%)	—	—
Pharmacological treatment			
Antiplatelet agents	104 (95.4%)	—	—
Statins	104 (95.4%)	—	—
Antihypertensive agents	103 (94.5%)	—	—
Proton-pump inhibitors	99 (90.8%)	—	—
Vasodilators	54 (49.5%)	—	—
Antidepressants/anxiolytics	22 (20.2%)	—	—
Analgesics	17 (15.6%)	—	—
Diuretics	13 (11.9%)	—	—
Oral anticoagulants	8 (7.3%)	—	—
Antiarrhythmic agents	1 (0.9%)	—	—
Baseline functional and BIA variables			
Duke Treadmill Score (points)	—	5.93 ± 3.70	−6.5–16.0
Dominant hand grip strength (kg)	—	34.9 ± 9.5	10.3–59.9
ECW/TBW ratio	—	0.432 ± 0.019	0.41–0.52

Abbreviations: BMI, body mass index; LVEF, left ventricular ejection fraction; NSTEMI, non-ST-elevation myocardial infarction; STEMI, ST-elevation myocardial infarction; PCI, percutaneous coronary intervention; CABG, coronary artery bypass graft; CR, cardiac rehabilitation; SD, standard deviation. Values are expressed as n (%)or means ± SD. ECW/TBW, extracellular-to-total body water ratio; IQR, interquartile range. ^1^ LVEF available in 105 participants; percentages refer to the total sample. ^2^ Data available in 108 participants; no complex arrhythmias were recorded.

**Table 2:** Phase angle (PhA) at baseline and post-intervention assessment by CR risk group.

CR Risk Group	n	Baseline PhA (Mean ± SD, °)	Post-Intervention PhA (Mean ± SD, °)	*p*-Value
Low risk	67	5.43 ± 0.65	5.52 ± 0.68	0.014 *†
Moderate risk	8	5.19 ± 0.53	5.24 ± 0.58	0.977
High risk	34	4.98 ± 0.81	4.99 ± 0.75	0.299 †
Total sample	109	5.27 ± 0.72	5.33 ± 0.74	0.016 *

Abbreviations: PhA, phase angle; CR, cardiac rehabilitation; SD, standard deviation. * *p* < 0.05. *p*-values correspond to the comparison between baseline and post-intervention PhA within each group (Wilcoxon signed-rank test); † At baseline, PhA was significantly higher in the low-risk than in the high-risk group (*p* = 0.002; Mann–Whitney U test).

**Table 3:** Spearman correlations between baseline phase angle (PhA) and functional, quality-of-life and clinical variables (N = 109).

Variable (Baseline Assessment)	n	r_s	r^2^ (%)	*p*-Value
SF-36 Physical Functioning	109	+0.291	8.5	0.002 **
Duke Treadmill Score (DTS)	109	+0.268	7.2	0.005 **
SF-36 Vitality	109	+0.265	7.0	0.005 **
SF-36 General Health	109	+0.217	4.7	0.024 *
Dominant hand grip strength (kg)	109	+0.196	3.8	0.041 *
Age (years)	109	−0.288	8.3	0.002 **
Charlson Comorbidity Index	109	−0.198	3.9	0.039 *
Fuster-BEWAT score	109	−0.210	4.4	0.028 *

Abbreviations: r_s, Spearman’s correlation coefficient; r^2^, coefficient of determination (percentage of shared variance); DTS, Duke Treadmill Score; SF-36, Short Form-36. * *p* < 0.05; ** *p* < 0.01. All correlations were considered at the bilateral level.

**Table 4:** Changes in functional and health variables between baseline and post-intervention assessment (N = 109).

Variable	Baseline (Mean ± SD)	Post-Intervention (Mean ± SD)	Δ	*p*-Value †
Phase angle (°)	5.27 ± 0.72	5.33 ± 0.74	+0.05	0.016 *
Duke (DTS)	5.93 ± 3.70	6.39 ± 4.26	+0.46	0.005 **
SF-36 Physical Functioning	73.1 ± 19.6	77.6 ± 18.4	+4.5	0.045 *
SF-36 Vitality	55.1 ± 20.9	60.7 ± 20.0	+5.6	0.003 **
SF-36 Bodily Pain	70.9 ± 26.1	77.2 ± 22.5	+6.3	0.019 *
SF-36 Mental Health	68.0 ± 19.3	72.4 ± 17.8	+4.4	0.034 *
Fuster-BEWAT (0–5)	2.73 ± 0.90	3.12 ± 0.98	+0.39	<0.001 ***
SBP (mmHg)	129.3 ± 18.4	121.6 ± 15.7	−7.7	<0.001 ***
Visceral fat (BIA units)	12.4 ± 3.9	12.1 ± 3.8	−0.3	<0.001 ***
Framingham (10-year risk, %)	29.4 ± 14.9	26.6 ± 13.5	−2.8	<0.001 ***

Abbreviations: Δ, baseline-to-post-intervention change; DTS, Duke Treadmill Score; SF-36, Short-Form-36; BEWAT, Fuster-BEWAT index; SBP, systolic blood pressure; BIA, bioelectrical impedance analysis; SD, standard deviation. † *p*-value of the Wilcoxon signed-rank test (bilateral comparison). * *p* < 0.05; ** *p* < 0.01; and *** *p* < 0.001.

**Table 7:** Discrimination and incremental values of phase angle (PhA) in logistic models for CR risk group (low vs. high risk; n = 101; 34 high-risk patients).

Model	AUC (95% CI)	CV-AUC	ΔAUC (*p*)	LR Test *p*	NRI (95% CI)	IDI (95% CI)
PhA alone	0.689 (0.574–0.803)	0.675	—	—	—	—
DTS alone	0.886 (0.814–0.958)	0.878	ref	ref	ref	ref
DTS + PhA	0.889 (0.819–0.960)	0.874	+0.004 (0.748)	0.028	0.34 (−0.07–1.03)	0.029 (−0.0004–0.135)
Clinical	0.689 (0.578–0.801)	0.622	ref	ref	ref	ref
Clinical + PhA	0.756 (0.649–0.864)	0.685	+0.067 (0.106)	0.003	0.64 (0.12–1.07)	0.083 (0.008–0.209)
Clinical + DTS	0.884 (0.813–0.955)	0.848	ref	ref	ref	ref
Clinical + DTS + PhA	0.910 (0.848–0.972)	0.864	+0.026 (0.117)	0.003	0.73 (0.07–1.39)	0.063 (0.0004–0.220)

Clinical model: age, sex, BMI and Charlson Comorbidity Index. AUC: apparent AUC with DeLong 95% CI, obtained, as in the original analysis, from L2-regularized logistic regression with standardized predictors (raw values for single predictors). CV-AUC: mean out-of-fold AUC over 20 repetitions of stratified 5-fold cross-validation. ΔAUC: difference versus the reference model immediately above (ref), with bootstrap *p*-value (2000 resamples). LR test: likelihood-ratio test of nested maximum-likelihood models. NRI: category-free net reclassification improvement; IDI: integrated discrimination improvement; 95% CIs from 2,000 bootstrap resamples. Abbreviations: AUC, area under the ROC curve; CI, confidence interval; CV, cross-validation; DTS, Duke Treadmill Score; LR, likelihood ratio; PhA, phase angle.

**Table 8:** ROC analysis: discriminatory capacity of phase angle (PhA) and Duke Treadmill Score for CR risk group classification (low vs. high risk; n = 101).

Model	AUC	95% CI	Sensitivity	Specificity
PhA alone	0.689	0.574–0.803	64.2%	76.5%
Duke alone	0.886	0.814–0.958	—	—
Duke + PhA (combined logistic)	0.889	0.819–0.960	—	—

Abbreviations: AUC, area under the ROC curve; CI, confidence interval; PhA, phase angle; CR, cardiac rehabilitation. AUC confidence intervals computed by the DeLong method. Sensitivity and specificity for PhA alone are reported at the optimal Youden cut-off (5.40°), with low risk as the positive class (PhA ≥ 5.40° → low risk). The combined model uses logistic regression (Duke + PhA); sensitivity/specificity are not reported, as no single optimal cut-off was pre-specified for the combined score.

**Table 9:** Multivariable linear regression of baseline phase angle (°) (n = 105).

Variable	β (°)	95% CI	*p*-Value
High risk (vs. low risk)	−0.486	−0.793 to −0.180	0.002 **
Moderate risk (vs. low risk)	−0.208	−0.708 to 0.291	0.414
Age (per year)	−0.026	−0.049 to −0.002	0.033 *
Male sex	+0.358	0.006 to 0.710	0.046 *
BMI (per kg/m^2^)	+0.041	0.004 to 0.078	0.031 *
Charlson Comorbidity Index (per point)	+0.039	−0.042 to 0.120	0.346
LVEF (per %)	−0.005	−0.035 to 0.025	0.735
Diabetes (pharmacologically treated)	−0.159	−0.424 to 0.106	0.240
Surgical revascularization (CABG)	+0.090	−0.206 to 0.385	0.552
Time from event to CR onset (per 100 days)	−0.034	−0.223 to 0.154	0.721

Ordinary least squares with heteroscedasticity-robust (HC3) standard errors; n = 105 participants with available LVEF; R^2^ = 0.304 (adjusted R^2^ = 0.230); variance inflation factors: 1.09–2.03. Sensitivity analyses: high-risk group β = −0.315° (95% CI: −0.567 to −0.062; *p* = 0.015) after additional adjustment for ECW/TBW (R^2^ = 0.642), and β = −0.495° (95% CI: −0.799 to −0.191; *p* = 0.001) after additional adjustment for multivessel disease and acute coronary syndrome as the index event. Abbreviations: BMI, body mass index; CABG, coronary artery bypass graft; CI, confidence interval; CR, cardiac rehabilitation; ECW/TBW, extracellular-to-total body water ratio; LVEF, left ventricular ejection fraction. * *p* < 0.05; ** *p* < 0.01.

## Data Availability

The data supporting the results of this study are available upon reasonable request to the corresponding author. The data are not publicly available for reasons of participant personal data protection, in accordance with the European Union General Data Protection Regulation (GDPR).

## Abbreviations

The following abbreviations are used in this manuscript:

ACS	Acute Coronary Syndrome
AUC	Area Under the ROC Curve
BIA	Bioelectrical Impedance Analysis
BMI	Body Mass Index
CABG	Coronary Artery Bypass Graft
CART	Classification and Regression Tree
CCI	Charlson Comorbidity Index
CI	Confidence Interval
COPD	Chronic Obstructive Pulmonary Disease
CPRU	Cardiac Prevention and Rehabilitation Unit
CR	Cardiac Rehabilitation
CV	Cross-Validation
CVD	Cardiovascular Disease
DTS	Duke Treadmill Score
ECW/TBW	Extracellular-to-Total Body Water Ratio
EWGSOP2	European Working Group on Sarcopenia in Older People 2
IDI	Integrated Discrimination Improvement
IQR	Interquartile Range
LR	Likelihood Ratio
LVEF	Left Ventricular Ejection Fraction
MET	Metabolic Equivalent
NRI	Net Reclassification Improvement
NSTEMI	Non-ST-Elevation Myocardial Infarction
OR	Odds Ratio
PCI	Percutaneous Coronary Intervention
PhA	Phase Angle
R	Electrical Resistance
ROC	Receiver Operating Characteristic
r_s	Spearman’s Correlation Coefficient
SBP	Systolic Blood Pressure
SD	Standard Deviation
SF-36	Short-Form-36 Health Survey
STEMI	ST-Elevation Myocardial Infarction
VIF	Variance Inflation Factor
Xc	Capacitive Reactance
